# Vaccination with an *Escherichia coli* F4/F18 Vaccine Improves Piglet Performance Combined with a Reduction in Antimicrobial Use and Secondary Infections Due to *Streptococcus suis*

**DOI:** 10.3390/ani12172231

**Published:** 2022-08-30

**Authors:** Frédéric A. C. J. Vangroenweghe, Mieke Boone

**Affiliations:** 1 Elanco Animal Health Benelux, BU Swine & Ruminants, 2018 Antwerpen, Belgium; 2 Unit of Porcine Health Management, Faculty of Veterinary Medicine, Department of Internal Medicine–Reproduction–Population Medicine, Ghent University, 9820 Merelbeke, Belgium; 3 Medivet DAP, 8020 Waardamme, Belgium

**Keywords:** *Escherichia coli*, vaccination, post-weaning diarrhea, piglet performance, antimicrobial use, *Streptococcus suis*

## Abstract

**Simple Summary:**

Post-weaning diarrhea (PWD) due to *Escherichia coli* (*E. coli*) remains a major cause of economic losses for the pig industry. Therapy to combat PWD typically consists of antibiotic treatment or supplementation of zinc oxide to the feed. The emergence of antimicrobial resistance and new EU regulations prompt the need for alternative control strategies, such as immunization. The aim of the field study was to evaluate the effect of an oral live non-pathogenic *E. coli* vaccine on piglet performance, health, and antimicrobial use. We compared 10 batches receiving a standard antimicrobial control treatment to 10 batches vaccinated with the oral *E. coli* vaccine. The vaccine-treated groups demonstrated a significant improvement in performance, mortality weight, and antimicrobial use. In addition, secondary infections due to *Streptococcus suis* in the second part of nursery were reduced, as indicated by the reduction in amoxicillin use. In conclusion, the present study demonstrates the efficacy of an oral live non-pathogenic *E. coli* vaccine for the active immunization of piglets against PWD under field conditions. Therefore, vaccination against PWD may be considered a valuable alternative for strengthening piglet performance while meeting the new EU requirements concerning the prudent use of antimicrobials in intensive pig production.

**Abstract:**

Post-weaning diarrhea (PWD) due to *Escherichia coli* (*E. coli*) remains a major cause of economic losses for the pig industry. Therapy to combat PWD typically consists of antibiotic treatment or supplementation of zinc oxide to the feed. The emergence of antimicrobial resistance to *E. coli* and new EU regulations prompt the need for alternative control strategies, such as immunization. The aim of the field study was to evaluate the effect of an oral live non-pathogenic *E. coli* vaccine on piglet performance, health, and antimicrobial use. We evaluated vaccination with an oral live non-pathogenic *E. coli* F4/F18 under field conditions in 10 consecutive batches against a standard antimicrobial treatment in 10 historical control batches. The vaccine-treated groups demonstrated a significant improvement in feed conversion rate, mortality weight, and antimicrobial use. From a general health perspective, secondary infections due to *Streptococcus suis* (*S. suis*) in the second part of nursery were markedly reduced, as indicated by the reduction in amoxicillin use. In conclusion, the present study demonstrates the efficacy of an oral live non-pathogenic *E. coli* vaccine for active immunization of piglets against PWD under field conditions. The vaccine-treated groups showed an improvement in several economically important performance parameters while reducing the overall antimicrobial use and infection pressure due to *S. suis*. Therefore, vaccination against PWD may be considered a valuable alternative for consolidating piglet performance while meeting the new EU requirements concerning the prudent use of antimicrobials in intensive pig production.

## 1. Introduction

Post-weaning diarrhea (PWD) in pigs is recognized as an economically important disease worldwide [1]. The most frequent characteristics of the disease condition are increased mortality, weight loss, retarded growth, increased treatment costs, higher use of antimicrobials, and more pronounced batch-to-batch variation [2,3,4,5,6,7,8]. Enterotoxigenic *Escherichia coli* (*E. coli*) (ETEC) has been identified as the most important cause of PWD. Two specific virulence factors typically characterize the ETEC pathotype, namely the presence of fimbrial adhesins, which mediate the attachment of bacteria to porcine intestinal enterocytes, and enterotoxins, which disrupt fluid homeostasis in the small intestine. Combined with a disruption in both the structure and absorptive function of the large intestine due to weaning-related stress [9,10], the infection with ETEC results in clinical signs of mild to severe diarrhea within a few days post-weaning. These clinical signs subsequently result in dehydration, loss of body condition (= disappearance of muscle volume), and mortality [11]. The adhesive fimbriae that most commonly occur in ETEC from piglets with PWD are F4 (formerly known as K88) and F18 [11]. Other fimbriae, such as F5 (K99), F6 (987P), and F41, rarely occur in *E. coli* isolates from PWD [11,12,13,14,15,16]. The main enterotoxins associated with porcine ETEC are heat-labile toxin (LT), heat-stable toxin a (STa), and heat-stable toxin b (STb). In some cases, the same pathogenic strain produces both enterotoxins and a Shiga toxin (Stx2e), which potentially results in both PWD and edema disease [11,14,15,16]. The disease is currently still controlled using antimicrobials, although the emergence of antimicrobial resistance in *E. coli* strains isolated from PWD over the last decades spurs the need for alternative control strategies [17,18,19,20,21].

Several alternative strategies to optimize intestinal health and decrease the incidence of PWD due to *E. coli* in post-weaned piglets have been explored [22,23,24]. In a first step, the inclusion of additional dietary fiber and a reduction in the crude protein levels in post-weaning diets have been examined as an effective nutritional strategy that may counteract the negative effects of protein fermentation in the pig gut [23,25,26,27]. Although specific fermentable carbohydrates combined with a reduced protein content could alter the microflora and fermentation patterns in the gastro-intestinal tract of post-weaned piglets, this favorable effect did not always result in an increased growth performance [28]. Other feeding strategies have focused on the feed consistency, thereby feeding a more coarsely ground meal to the post-weaned piglets [29]. Coarsely ground meal alters the physico-chemical conditions at the level of the stomach and increases the concentrations of organic acids, which result in a lower pH of the feed. This promotes the growth of anaerobic lactic acid bacteria, thereby reducing the survival of pathogenic *E. coli* during passage through the stomach [29]. Other factors, such as fermentation of undigested dietary protein and endogenous proteins in the large intestine, may produce putative toxic metabolites that impair epithelial integrity and promote enteric disorders, such as PWD [30]. The addition of probiotics to the diet, which may change the fermentation profile and thus promote gut health, may also influence the incidence and severity of PWD [31]. Furthermore, medium chain fatty acids (MCFAs) help to neutralize bacterial metabolites in the small intestine, thereby improving gut health overall as well as the challenge with pathogenic *E. coli* in particular [32]. 

Several decades ago, in the early 1980s, multiple studies on the supply of zinc to post-weaned piglets were performed. The supplementation of dietary zinc oxide (ZnO) has been proven to play an important role in the prevention and healing of PWD in several nutritional studies [33]. Based on these data, ZnO was approved for prevention and control of PWD at levels up to 3000 parts per million (ppm) through the feed for a maximum period of 14 days post-weaning. This approach implied a serious reduction in antimicrobial use related to the treatment of PWD. However, the Committee for Veterinary Medicinal Products (CVMP) has recently decided that the use of ZnO in post-weaning diets to prevent PWD should be phased out by 2022 at the latest throughout the EU [34]. 

Therefore, other preventive strategies toward PWD due to ETEC have recently been explored [21,35]. For an *E. coli* vaccination against PWD due to F4- and F18-ETEC, the prerequisite is that active mucosal immunity against both F4 and F18 is mounted prior to exposure to the pathogenic F4- and F18-ETEC strains. The activated mucosal immunity results in local production of F4- and/or F18-specific secretory IgA antibodies, which prevent pathogenic F4- and F18-ETEC from attaching to the intestinal F4- and F18-receptors, thus reducing the appearance of clinical signs of PWD [35]. Recently, vaccination with an oral live non-pathogenic *E. coli* F4 or *E. coli* F4 and F18 vaccine has demonstrated efficacy against PWD due to F4-ETEC and F4- and F18-ETEC [36,37,38,39,40]. Immunization against the F4- and F18-ETEC pathogens decreased both the severity and duration of PWD’s clinical signs as well as fecal shedding of F4- and F18-ETEC [36,37]. Moreover, piglets vaccinated with an *E. coli* F4 vaccine had increased weight gain [36]. A recent summary of 10 field trials in Belgium and the Netherlands demonstrated improved piglet performance (feed conversion rate, FCR) combined with similar average daily weight gain and reductions in mortality and antimicrobial use following vaccination with an oral live non-pathogenic *E. coli* F4/F18 vaccine [40]. 

Besides PWD due to F4- and F18-ETEC, *S. suis* is one of the most important bacterial swine pathogens affecting post-weaned piglets. Streptococcal disease in this animal category is mainly characterized by meningitis, arthritis, and sudden death [41]. Co-infection with some viral (PRRS, PCV-2 and IAV-S) and bacterial (*E. coli*, *Actinobacillus pleuropneumoniae*) pathogens can significantly influence the severity of *S. suis*-associated diseases and may be the key to understanding how the infection behaves in the field [41]. Even when the carrier rate of *S. suis* is high, the incidence of disease can vary from period to period and is usually lower than 5%, mainly due to prophylactic or metaphylactic measures. This approach is in contrast with the current trend to reduce antimicrobial use in animals and humans as much as possible to prevent further development of antimicrobial resistance [41]. Although the pathogenesis mechanisms of *S. suis* infection are poorly understood, many virulence factors have already been identified that play a role in the crucial steps of infection development [42]. Following colonization of the host, progression through mucous membranes (tonsils, intestine, etc.) using its suilysin, a hemolysin with cytotoxic properties, results in the *S. suis* bacteria spreading into the blood stream [42]. There, the bacteria can escape the host’s immune response and, finally, cross the blood–brain barrier, which results in meningitis. 

Here, we report results demonstrating the efficacy of an oral live non-pathogenic *E. coli* F4/F18 vaccine (Coliprotec^®^ F4/F18; Elanco GmbH, Heinz-Lohmann-Str. 4, 27472 Cuxhaven, Germany) for active immunization of piglets against PWD caused by F4-ETEC in 10 consecutive batches against a control group treated with the standard therapeutic approach using antimicrobial therapy. We evaluated piglet performance, piglet health related to secondary infections due to *Streptococcus suis*, and overall antimicrobial treatment during the post-weaning period.

## 2. Materials and Methods

### 2.1. Experimental Farm Description

The field trial was performed on a conventional farrow-to-finish pig farm with 1000 TN70 sows in Flanders (Belgium). The farm was managed in a 4-week batch management system with 160 sows per production batch. This management approach has been shown to improve the health status for several respiratory pathogens [43]. Sows were vaccinated once at 3 weeks prior to farrowing with a commercial *E. coli–C. perfringens–C. novyi* vaccine (Suising; Hipra, Amer, Spain). Gilts were vaccinated twice at 6 and 3 weeks prior to farrowing with the same commercial vaccine. Piglets were weaned at 22 days of age and housed in specifically equipped post-weaning facilities, where they were raised for 6.5 to 7 weeks (46 to 50 days post-weaning). The post-weaning facility was divided into five compartments of 28 pens each, which could house 20 post-weaned piglets. Dry feeders with two waterers, one on each side, were in the pen division, thus feeding two pens with a total of 40 piglets. The pens were further equipped with fully slatted plastic floors and were heated with hot water tubes located in the ventilation duct. Ventilation was performed through one ventilation fan evacuating the air into a central ventilation duct, and fresh air entered each compartment indirectly through the central corridor after being pre-heated if necessary.

### 2.2. ETEC Diagnosis and Characterization at the Experimental Farm

The farm suffered for several years from PWD outbreaks due to ETEC in each consecutive batch. The farm was selected following ETEC diagnostics during the post-weaning period. Therefore, untreated piglets (*n* = 10) with typical clinical signs of PWD, such as watery diarrhea, a thin belly, and signs of dehydration, were sampled using rectal swabs (Sterile Transport Swab Amies with charcoal medium; Copan Italia S.p.A., Brescia, Italy). All sampled piglets were between 3 and 5 days post-weaning. The diagnostic samples were sent to the laboratory (IZSLER, Brescia, Italy) under cooled conditions for further processing. 

The specimens were processed using standard procedures for isolation and characterization of intestinal *E. coli* [21]. Briefly, samples were plated on selective media and on tryptose agar medium supplemented with 5% defibrinated ovine blood and incubated aerobically overnight at 37 °C. Hemolytic activity was evaluated, and single coliform colonies were further characterized.

DNA samples were prepared from one up to five hemolytic and/or non-hemolytic *E. coli* colonies and used to perform a multiplex PCR for the detection of fimbrial and toxin genes, including those encoding for F4 (K88), F5 (K99), F6 (987P), F18, F41, LT, STa, STb, and Stx2e, but not discriminating between F4ab, F4ac, and F4ad. The methodology used for identification of these virulence genes has been described previously [44]. All collected samples were positive for F18 in combination with STa and STb. No other virulence factors could be detected. The identified F18-ETEC strain was resistant to several important antimicrobials applied in the treatment of PWD, such as apramycin, colistin, flumequine, gentamycin, trimethoprim-sulfa, and spectinomycin (Table 1).

### 2.3. Diagnostic Approach of Mortality in the Second Phase of the Nursery Period Vaccine

Mortality observed during the second phase of the nursery period was characterized by acute death with no preceding clinical signs or subacute death preceded by symptoms of meningitis. Several typical clinical cases were submitted to a diagnostic laboratory (DGZ-Vlaanderen, Torhout, Belgium) for necropsy and further analysis. All cases were confirmed positive for *S. suis* with a resistance to several antimicrobials applied during treatment of streptococcal meningitis, such as tetracycline, doxycycline, and lincomycin (Table 1). 

### 2.4. Vaccination with an Oral Live Non-Pathogenic E. coli F4/F18 Vaccine

To vaccinate piglets at least 7 days prior to the clinical signs, in order to mount sufficient protective local immunity in the gut [36], piglets were vaccinated during the suckling period at 18 days of age (4 days prior to weaning),. The live non-pathogenic *E. coli* F4/F18 vaccine (Coliprotec^®^ F4/F18; Elanco GmbH, Heinz-Lohmann-Str. 4, 27472 Cuxhaven, Germany) has a rapid onset of immunity (OOI) of only 7 days and a duration of immunity (DOI) of 21 days post-vaccination, which covers the most important critical period for the occurrence of PWD [1]. An efficacy trial using an experimental *E. coli* F4 challenge at 3 days post-vaccination demonstrated a reduction in both the severity and duration of PWD and a reduction in fecal shedding of pathogenic F4-ETEC [36]. The vaccine was administered orally through the water bowls in the farrowing room, mixing 14 vaccine doses into 1 L of cold water for 1 litter of suckling piglets. No antibiotics were administered to piglets from 15 days of age onwards to avoid interference from the development of protective local immunity by the *E. coli* F4/F18 vaccine during the 7 days following vaccination. 

### 2.5. Experimental Design and Treatment

The field trial was carried out between 16 November 2020, when the first control group was enrolled, and 20 June 2022, when the last vaccine-treated group was finalized. Due to the practical setting, it was not possible to run control and vaccine-treated groups concurrently in one batch of weaned piglets. Moreover, since vaccination with the *E. coli* F4/F18 vaccine reduced the excretion of pathogenic *E. coli* bacteria in the vaccine-treated group [36], alternating between control and vaccine-treated groups was not possible either, since this would affect the infection pressure in the control groups. Therefore, we worked with a historical control group of 10 consecutive batches, followed by a treatment group of 10 consecutive batches receiving the oral live non-pathogenic *E. coli* F4/F18 vaccine, as described previously. 

### 2.6. Performance and Health Parameters

The following performance parameters were collected during the trial: number of piglets enrolled in each batch (*n*), number of piglets moved to the next phase (*n*), date of weaning and end of nursery period, piglet body weight (BW; kg/piglet) at d0 and at the end of the nursery period (d46-50) (kg/piglet), feed intake during the nursery period (kg/piglet), and mortality (*n*, %) with weight at death (kg).

The following health parameters were collected during the trial: amount of active ingredient prescribed and administered to the specific batch of piglets, type of antimicrobial substance used, and cost of treatment.

Based on these parameters, the following performance and health indicators were calculated: number of days in nursery (d), average daily weight gain (ADWG, g/d), average daily feed intake (ADFI, g/d), feed conversion rate (FCR, kg feed/kg weight gain), percentage of piglets transferrable to the next phase (%), treatment cost (€/piglet), and treatment incidence over a 100-day period in the nursery (TI_100_). 

### 2.7. Statistical Analysis

For statistical analysis, JMP 15.1.0 Statistical Software (SAS, Marlow, UK) was applied. For the continuous data, the effect of the treatment on the different outcome parameters was assessed using a t-test with pooled standard deviations. All tests were performed at the nominal level of 5%.

## 3. Results

### 3.1. Piglet Weight and Average Daily Weight Gain

The total BW of the weaned piglet was not significantly (*p* = 0.386) different between the control group (17,475 ± 921 kg) and the vaccine-treated group (17,895 ± 1088 kg). The total sold piglet weight was not significantly (*p* = 0.414) different between the control group (60,732 ± 1325 kg) and the vaccine-treated group (61,183 ± 1567 kg). The average percentage of sold piglets per batch was numerically, but non-significantly (*p* = 0.094), higher in the vaccine-treated group (97.1 ± 0.2%) than the control group (96.5 ± 0.4%) (Table 2). The average BW at weaning was not significantly (*p* = 0.406) different between the control group (6.61 ± 0.22 kg) and the vaccine-treated group (6.53 ± 0.23 kg). The average BW at selling was not significantly (*p* = 0.063) different between the control group (23.97 ± 0.41 kg) and the vaccine-treated group (23.18 ± 0.26 kg) (Table 2). The period in nursery was significantly (*p* = 0.027) shorter in the control group (46.7 ± 0.5 d) than the vaccine-treated group (49.6 ± 0.6 d). The total weight gain (WG) from weaning to selling was similar in both treatment groups (17.36 ± 0.50 kg in the control group vs. 16.65 ± 0.34 kg in the vaccine-treated group, respectively) (*p* = 0.129). Nevertheless, the ADWG was significantly (*p* = 0.008) higher in the control group (375 ± 12 g) than the vaccine-treated group (336 ± 6 g) (Table 2).

### 3.2. Piglet ADFI and FCR

The total feed intake (FI) per sold piglet (considering the feed consumption of all dead piglets during the nursery period) was higher, but not significantly (*p* = 0.052) different, in the control group (28.98 ± 1.00 kg) than the vaccine-treated group (26.84 ± 0.56 kg). The ADFI was significantly (*p* = 0.008) lower in the vaccine-treated group (540 ± 9 g/d) than the control group (625 ± 27 g/d) (Table 2). The FCR, calculated as the amount of feed needed for each kg of weight gain, was significantly (*p* = 0.041) better in the vaccine-treated group (1.61 ± 0.02) than the control group (1.67 ± 0.02) (Table 2).

### 3.3. Piglet Number: Weaned, Sold, and Mortality

A total of 10 batches of control piglets and 10 batches of vaccine-treated piglets were enrolled in the field study. On average, 2632 (±63) and 2720 (±98) piglets were weaned per batch in the control and vaccine-treated group, respectively (*p* = 0.181). At selling, an average of 2541 (±68) and 2589 (±68) piglets per batch were eligible to move to the next phase in the control and vaccine-treated groups, respectively (*p* = 0.157) (Table 2).

A total of 91 (±9) and 79 (±6) piglets died per batch during the entire nursery period in the control and vaccine-treated groups, respectively. This number was not significantly different between both treatment groups (*p* = 0.133). In the control group, 3.52 (±0.38) % of the piglets died during the nursery period, whereas in the vaccine-treated group 2.90 (±0.25) % died during the same period. The mortality rate (expressed as a percentage) was not significantly different between both groups (*p* = 0.094) (Table 2). Nevertheless, the lightest 10% of the piglets (weaning weight below 3.5 kg) accounted for approximately 90% of all mortalities in both groups (data not shown). 

The average weight of dead piglets was significantly (*p* = 0.00013) lighter in the vaccine-treated group (5.46 ± 0.42 kg) than the control group (7.80 ± 0.26 kg) (Table 2). 

### 3.4. Antimicrobial Use

**Figure 1:**
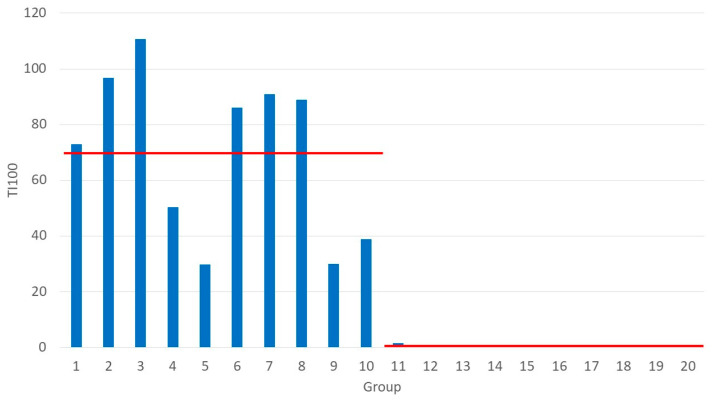
Treatment incidence 100 (TI_100_) calculated as number of treatment days per 100 days in the nursery period. Group 1–10 are control piglets, and group 11–20 are vaccine-treated piglets. Red lines indicate the average TI_100_ for both treatment groups: 69.43 ± 9.44 for the control group and 0.13 ± 0.13 for the vaccine-treated group. TI_100_ is significantly (*p* = 0.000022) different between both treatment groups.

The antimicrobial use, expressed as TI_100_, or the number of days on treatment for every 100 days in nursery, was significantly (*p* = 0.000022) higher in the control group (69.42 ± 9.44 d) than the vaccine-treated group (0.13 ± 0.13 d) (Figure 1). This represents a 99.8% reduction in antimicrobial use between the control and the vaccine-treated group. Moreover, in the vaccine-treated batches, 9 months could note a TI_100_ of zero, whereas none of the control batches could represent this result (Table 2). From Figure 1, it can be observed that the TI_100_ in the control group varied between 29.7 and 110.7 days, whereas in the vaccine-treated group the TI_100_ was only 1.32 in the first month after implementation of the vaccination program against PWD and continued to remain at zero for the subsequent nine batches (Figure 1).

From the perspective of treatment costs, a significant reduction (*p* = 0.0086) was observed between the control group (€ 0.36 ± 0.11) and the vaccine-treated group (€ 0.05 ± 0.02). This represents an 86.1% reduction in the antimicrobial treatment costs between the control and vaccine-treated group (Table 2).

Details on the different antimicrobials used for the treatment of post-weaning diarrhea due to F18-ETEC are given in Table 3. In the control, an average of 5.6 kg of antimicrobial products, including lincomycin-spectinomycin, colistin, doxycycline, trimethoprim-sulfa, and apramycin, was used in every batch. In contrast, no antimicrobials intended to treat the clinical problems of PWD were prescribed or used following the implementation of the oral live non-pathogenic *E. coli* vaccine in the farm (Table 3). Thus, antimicrobial use for the treatment of PWD showed a 100% reduction following vaccination.

### 3.5. Secondary Infections with S. suis

From a clinical point of view, a clear reduction in the prevalence in streptococcal meningitis due to *S. suis* may be observed by both the farm owner and the farm veterinarian, responsible for clinical surveillance and follow-up of the farm. This observation can be confirmed by both the significant (*p* = 0.0013) reduction in the weight of dead piglets between the control group (7.80 ± 0.26 kg) and the vaccine-treated group (5.46 ± 0.42 kg) and by the 91.5% reduction in the amount of amoxicillin used for the treatment of streptococcal meningitis in the second phase of the nursery period between the control group (59 kg over 10 batches) and the vaccine-treated group (5 kg only used in the first vaccinated batch) (Table 3). In addition, the kinetics of the TI_100_ per batch clearly demonstrates that after the first vaccinated batch, the TI_100_ categorically drops to zero and remains at that level for the entire remaining study period (Figure 1). 

## 4. Discussion

The current field study on the vaccination of piglets pre-weaning to protect against PWD due to F18-ETEC clearly demonstrates that the overall technical performance was not significantly different between both treatment groups, considering that the control group had a significantly higher antimicrobial use than the vaccine-treated group. Several economically important performance parameters, such as ADFI, FCR, antimicrobial treatment cost, and TI_100_, were significantly improved in the vaccine-treated group as compared to the control group. 

The total WG in nursery was only 600 g lower in the vaccine-treated group over the entire nursery period of about 50 days. Several factors might explain this slight, but non-significant, difference. In the vaccine-treated groups, about 90 supplementary piglets were weaned per batch, which may explain the lighter weaning weight of 90 g per piglets in this group. Another important factor that may have contributed to the slightly lower total WG in the nursery period was the significant reduction in antimicrobial use observed both for the treatment and control of PWD in the first phase of nursery and streptococcal meningitis in the second phase of nursery (Table 3), which resulted in a drastic decrease in the TI_100_ from 69.43 d to 0.13 d. It has been shown that even therapeutic use of antimicrobials results in an improvement in animal growth efficiency through inhibition of the normal gut microbiota, eventually leading to increased nutrient utilization and reduction in the maintenance cost of the gastro-intestinal system [45].

For FCR, an improvement of 0.06 in piglet performance represents an approximate economic advantage of about 3.7 eurocent per kg gain—at the current average market value of EUR 620 per ton of post-weaning piglet feed—or 1.05 kg less feed per piglet for the same post-weaning WG. Feed cost should be considered the most important economic aspect in piglet production; the above calculated benefit may already have a significant impact on the net farm’s income during the post-weaning production phase. In fact, the significantly lower ADWG (−39 g/d) in the vaccine-treated group is therefore largely compensated for by a significantly lower ADFI (−85 g/d), which could result in this significant improvement in the FCR in the vaccinated piglets. These results are in accordance with a recent analysis of 10 field trials in Belgium and the Netherlands in which a similar significant improvement in FCR could be observed [40]. In contrast, the same study demonstrated a slight, but non-significant, improvement in ADWG over the 10 different field studies, which were carried out under different field conditions.

The current field study was carried out using a historical control group, which could be considered a minor negative aspect. However, from a practical point of view, the current farm did not allow us to run concurrent treatment groups at the same time due to several practical constraints. Although several compartments were available in the nursery phase, only one automated feed line was available to distribute the feed to all compartments. In addition, the construction of the barn did not efficiently allow for separation of both treatment groups with a sufficient level in internal biosecurity to omit the spread of pathogens between both treatment groups. A third practical issue was that the piglets had to be vaccinated pre-weaning, since the onset of immunity of the oral live non-pathogenic *E. coli* vaccine is 7 days, and piglets of different litters are commingled according to their weight and general condition at weaning. Changes in these day-to-day routine management aspects would complicate the set-up and performance of this practical field trial and might lead to involuntary and non-detectable errors that may have blurred the results and conclusions. Use of the current study design allowed us to change only one specific parameter—i.e., vaccination of piglets prior to weaning—and evaluate the effects of this implemented strategy with changes to any other management practices. 

In Belgium, antimicrobial use at the farm and animal category level (sows, piglets, fattening pigs) is registered in a central database (Ab Register, www.abregister.be (accessed on 15 July 2022)) by the farm veterinarian on a quarterly basis. Based on these registrations and the individual delivery documents of the antimicrobial products, we were able to analyze the TI_100_ and details of all administered products for both clinical conditions (PWD and streptococcal meningitis) at the farm. The current study demonstrated a drastic decrease in antimicrobial use following implementation of an oral live non-pathogenic *E. coli* vaccine in piglets to prevent the clinical signs of PWD due to F18-ETEC. Indeed, both the overall TI_100_ and the more detailed data per batch showed a marked decrease in antimicrobial use following vaccination. The average amount of antimicrobial prescribed and used for the treatment of PWD decreased from 5.6 kg per batch to 0.0 kg, which resulted in nine consecutive batches with a TI_100_ of zero. The only antimicrobial used following vaccination was amoxicillin in the first vaccinated batch for the treatment and control of streptococcal meningitis due to *S. suis*. However, treatment of this clinical condition was not needed from the second vaccinated batch onward. These results are in accordance with previous studies using the *E. coli* vaccine [38,39,40], in which similar reductions of approximately 80–95% could be observed. In the current study, an 86.1% reduction in antimicrobial treatment costs and a 99.8% reduction in TI_100_—total number of treatment days per 100 days in nursery—can be registered. 

Mortality data were recorded in detail to keep track of the number of dead piglets and the weight of the piglets. A non-significant reduction in mortality (−0.62%) was observed in the vaccine-treated group as compared to the control group. Of the total number of weaned piglets (26,318 and 27,195 piglets in the control and vaccine-treated group, respectively), this reduction in mortality resulted in an increase in the number of piglets that could move to the next phase (25,405 and 26,409 piglets in the control and vaccine-treated group, respectively), which may also be considered an important economic benefit following vaccine implementation. 

Another remarkable aspect of the mortality data, besides the slightly lower mortality in the vaccine-treated group, is that the average BW of dead piglets is significantly lower (−2.36 kg per dead piglet) in the vaccine-treated group than in the control group. In practice, it is known that light-weight piglets predominantly die in the first 2 weeks post-weaning from PWD due to F18-ETEC and other problems related to livability due to their low weaning weight, whereas heavier piglets predominantly die later during the nursery period due to streptococcal meningitis due to *S. suis*. The decrease in the average BW of dead piglets was mainly due to the decrease in second phase mortality related to these clinical problems with streptococcal meningitis, which can be confirmed by the antimicrobial consumption data of amoxicillin in the vaccine-treated group. Only in the first vaccinated batch was amoxicillin needed for the treatment and control of streptococcal meningitis due to *S. suis*. Thereafter, no amoxicillin was prescribed or used in the vaccine-treated group. A potential explanation may be found in the improved intestinal integrity following an oral live non-pathogenic *E. coli* vaccination, which may result in a lowered opportunity for *S. suis* to colonize the host and subsequently progress through the intestinal mucous membranes using its suilysin to finally spread through the blood to the meninges after passing the blood–brain barrier [42]. Although co-infection patterns of *S. suis* as a secondary pathogen following initial viral or bacterial infections have been reported [41], little is known about the interaction between PWD due to ETEC and subsequent clinical episodes of streptococcal meningitis due to *S. suis*. Moreover, induction of this specific combination of co-infection between PWD due to ETEC and streptococcal meningitis due *S. suis* is difficult to reproduce under experimental conditions. 

## 5. Conclusions

The current field study reports the efficacy of an oral live non-pathogenic *E. coli* vaccine for the active immunization of piglets against PWD due to F18-ETEC under field conditions. Vaccine-treated groups showed improvements in several economically important performance parameters while reducing the overall antimicrobial use and infection pressure due to *S. suis*. Therefore, vaccination against PWD may be considered a valuable alternative for strengthening piglet performance while meeting the new EU requirements concerning the prudent use of antimicrobials in intensive pig production.

## Figures and Tables

**Table 1:** Diagnostic laboratory results on isolation, identification, and antimicrobial resistance profile of the *Escherichia coli* strain involved in post-weaning diarrhea and the secondary clinical problem of acute mortality due to *Streptococcus suis* meningitis. Gray-colored blocks indicate the absence of relevant information.

Pathogen	*Escherichia coli*	*Streptococcus suis*
Culture morphology	Hemolytic	
*Adhesins/fimbriae*		
F4 (K88)	Negative	
F18	Positive	
*Toxins*		
STa	Positive	
STb	Positive	
LT	Negative	
Stx2e	Negative	
*Pathotype*	F18-ETEC	
*Virotype*	F18 STa STb	
*Antimicrobial resistance profile*		
Amoxicillin	Resistant	Sensitive
Apramycin	Resistant	
Cefalexin	Intermediary	Sensitive
Cefquinome	Sensitive	Sensitive
Ceftiofur	Sensitive	Sensitive
Colistin	Resistant	
Doxycyclin		Resistant
Enrofloxacin	Sensitive	Sensitive
Erythromycin		Resistant
Florfenicol	Sensitive	Sensitive
Flumequine	Resistant	
Gentamycin	Resistant	
Kanamycin	Resistant	Resistant
Lincomycin		Resistant
Marbofloxacin	Sensitive	
Paromomseycin	Sensitive	
Penicillin		Sensitive
Spectinomycin	Resistant	
Sulfa-trimethoprim	Resistant	Sensitive
Tetracyclin	Resistant	Resistant
Tylosin		Resistant

**Table 2:** Performance data of a comparative field trial with a historical control group with standard antimicrobial treatment and a vaccine-treated group using Coliprotec F4F18 (Elanco) on a farm with clinical problems of post-weaning diarrhea due to F4-ETEC. Significant differences are indicated by the superscript letter and their *p*-value.

Performance Parameter	Control	Vaccine	*p*-Value
Number of groups	10	10	-
Number of weaned piglets (±SEM)	2632 ± 63	2720 ± 98	0.181
Total BW of weaned piglets (kg ± SEM)	17,475 ± 921	17,895 ± 1088	0.386
Average BW at weaning (kg ± SEM)	6.61 ± 0.22	6.53 ± 0.23	0.406
Number of sold piglets (±SEM)	2541 ± 68	2641 ± 69	0.157
Percentage sold piglets (±SEM)	96.48 ± 0.4	97.10 ± 0.2	0.094
Total BW of sold piglets (kg)	60,732 ± 1325	61,183 ± 1567	0.414
Average BW at selling (kg ± SEM)	23.97 ± 0.41	23.18 ± 0.26	0.063
Total WG (kg ± SEM)	17.36 ± 0.50	16.65 ± 0.34	0.129
# days in nursery (d ± SEM)	46.7 ± 1.2 ^a^	49.6 ± 0.6 ^b^	0.027
Mortality (# ± SEM) (% ± SEM)	91 ± 9	79 ± 6	0.133
	3.52 ± 0.38	2.90 ± 0.25	0.094
BW of dead piglets (kg ± SEM)	7.80 ± 0.26 ^a^	5.46 ± 0.42 ^b^	0.00013
FI per sold piglet (kg ± SEM)	28.98 ± 1.00	26.84 ± 0.56	0.052
ADFI (g ± SEM)	625 ± 27 ^a^	540 ± 9 ^b^	0.008
ADWG (g ± SEM)	375 ± 15 ^a^	336 ± 6 ^b^	0.018
FCR (kg feed/kg gain ± SEM)	1.67 ± 0.02 ^a^	1.61 ± 0.02 ^b^	0.041
Antimicrobial treatment cost per piglet (€ ± SEM)	0.36 ± 0.11 ^a^	0.05 ± 0.02 ^b^	0.0086
Reduction in cost of antimicrobial treatment		86.1%	-
TI_100_ (d ± SEM)	69.43 ± 9.44 ^a^	0.13 ± 0.13 ^b^	0.000022
Reduction in antimicrobial use (%)		99.8%	-
Month with TI_100_ = 0	0	9	-

**Table 3:** Summary of active ingredients of antimicrobials (expressed in kg of commercial product; including calculated total amount and average amount of product per group) administered for treatment of *S. suis* and *E. coli* in the 10 batches of the control group and 10 batches of the vaccine-treated group.

Pathogen	*S. suis*			*E. coli*						
		Total Amount	Average per Group						Total Amount	Average Per Group
Active ingredient	Amoxicillin			Lincomycin- spectinomycin	Colistin	Doxycycline	Trimethoprim-sulfa	Apramycin		
Control	59	59	5.90	3	4	27	4	12.6	50.60	5.06
Vaccine	5	5	0.50	0	0	0	0	0	0.00	0.00

## Data Availability

The dataset analyzed during the current study is available from the corresponding author on reasonable request.
